# Temporal specialization of the neural memory system: common and distinct neural correlates of recent and remote memory retrieval

**DOI:** 10.3389/fnimg.2025.1584849

**Published:** 2025-06-10

**Authors:** Rudolf Krug, Marko Rajkovic, Marco Caviezel, Else Schneider, Stefan Borgwardt, Annette Beatrix Bruehl, Undine Lang, Christoph Linnemann, Tobias Melcher

**Affiliations:** ^1^Center of Old Age Psychiatry, University Psychiatric Clinics (UPK), University of Basel, Basel, Switzerland; ^2^Transfaculty Research Platform Molecular and Cognitive Neuroscience, University of Basel, Basel, Switzerland; ^3^Center of Affective, Stress and Sleep Disorders (ZASS), University Psychiatric Clinics (UPK), University of Basel, Basel, Switzerland; ^4^Department of Clinical Research (DKF), University of Basel, University Psychiatric Clinics (UPK), Translational Neurosciences, Basel, Switzerland; ^5^Translational Psychiatry Unit (TPU), Department of Psychiatry and Psychotherapy, University of Lübeck, Lübeck, Germany; ^6^University Psychiatric Clinics (UPK), University of Basel, Basel, Switzerland

**Keywords:** fMRI, remote memory, recent memory, anterior temporal lobe, anterior insular cortex (aIC), posterior midline region (PMR), associative memory (AM)

## Abstract

**Introduction:**

Associative memory (AM) is the most basic and common memory form. It constitutes the foundation of the declarative memory system, including all semantic and episodic memory processes. However, despite numerous studies, recent and remote memory retrieval processes in AM still need further elucidation.

**Methods:**

Here, we investigated the neurofunctional correlates of recent and remote-related AM retrieval using associative face-name pairs of famous and non-famous individuals in a population of young, healthy adults (*N* = 23; mean age = 23.39 years). Particular interest was placed on the prominent anterior temporal lobe (ATL) found during recent and remote memory, including the right anterior insular (aIC) cortex and posterior midline region (PMR) previously observed during associative memory retrieval.

**Results:**

The results of the present study revealed significant bilateral activation in the anterior parts of the STG as subdivision of the ATL during recent and remote memory retrieval. In addition, bilateral aIC activation was observed exclusively during recent memory retrieval, while PMR and ventromedial prefrontal cortex (vmPFC) activity was found only during remote memory retrieval.

**Discussion:**

Thus, the present results corroborate the ATL's role as a common hub not only for AM retrieval but also for recent and remote memory processes. In addition, the recent and remote memory retrieval systems also appear to engage distinct neurofunctional networks to enable successful retrieval of contingent face-name pairs.

## 1 Introduction

Associative memory (AM) has been conceived as an essential memory function that encompasses two interrelated aspects. Firstly, the formation, and secondly, the retrieval of a cross-linking between two or more initially unrelated mental representations, e.g., a name and an unfamiliar face (Kandel and Pittenger, 1999; Naveh-Benjamin and Mayr, 2018). This phenomenon becomes especially noticeable in everyday scenarios of memorisation, illustrated by the retention or possible oversight of an individual's name (Caviezel et al., 2020). These associative mental representations can be qualitatively distinguished from each other based on their respective retention duration in the human memory system. More specifically, associative mental representations can be divided into recently acquired and remotely acquired. Remote memory involves retrieving associations from a more distant past, typically spanning years or decades. In contrast, recent memory refers to retrieving information within minutes to months after initial encoding (Kreutzer et al., 2011).

Traditionally, AM has been functionally attributed to the declarative or relational memory system, which contains mental representations of facts, i.e., semantic memory, and biographical events, i.e., episodic memory (Suzuki, 2008). In recent decades, extensive research has been conducted to investigate the underlying neurofunctional anatomy of AM, both in healthy (Caviezel et al., 2020; Huijbers et al., 2012; Vannini et al., 2011) and clinical populations (Naveh-Benjamin and Mayr, 2018; Poch et al., 2021). Traditionally, the medial temporal lobe (MTL), which encompasses the hippocampus (HPC) and its neighboring cortical regions, has been highlighted as a central hub of AM functioning during memory encoding and retrieval (Eichenbaum, 2004; Knierim, 2015; Poch et al., 2021). However, the emergence of key neuropsychological concepts, such as the standard consolidation theory, has led researchers to believe that the importance of the HPC for retrieving memory representations diminishes over time, additionally, recent evidence suggests that the cortex's involvement increases once lasting memory connections have been established through (re-) consolidation (Gilboa and Moscovitch, 2021). This includes, most prominently, the posterior midline region (PMR), comprising the precuneus and the posterior and middle cingulate cortex (PCC/MCC), and the ventromedial prefrontal cortex (vmPFC) (Bonnici and Maguire, 2018; Eichenbaum, 2004; Knierim, 2015). These regions are also considered part of the default mode network (DMN), which is typically active during resting states as well as during the retrieval of distant and episodic memories (Daselaar et al., 2009).

The neurofunctional correlates of AM are not only determined by the time interval between the initial learning and subsequent retrieval of an acquired memory representation but can also be influenced by the type of memory that is retrieved at a given time, such as semantic or episodic memory representations. In this context, the anterior temporal lobe (ATL), has emerged in the literature as an amodal semantic hub in the integration and activation of semantic representations across all sensory modalities, e.g., visual and acoustic, and semantic categories, e.g., formal, conceptual, and linguistic (Patterson et al., 2007; Pisoni et al., 2020). Additionally, the involvement of the ATL in semantic processing occurs bilaterally and has been proposed to exhibit particularly robust activity during the recognition of well-known individuals, i.e., famous people's faces (Brambati et al., 2010; Patterson et al., 2007; Poch et al., 2021). Therefore, semantic memory retrieval relies especially on cortical regions, such as the ATL encompassing the anterior divisions of the inferior, middle and the superior temporal gyrus (MTG/STG) (Bonner and Price, 2013; Brambati et al., 2010; Gilboa and Moscovitch, 2021; Pisoni et al., 2020; Yagishita et al., 2008).

Another brain region that recently has sparked interest regarding the retrieval of AM is the anterior insular cortex (aIC) which previously has been mainly associated with the detection of salient events and regulatory processes that require higher cognitive control (Engström et al., 2015; Ghahremani et al., 2015). In a recent fMRI investigation bilateral activation of the aIC was found during semantic and episodic memory retrieval (Vatansever et al., 2021). Similar evidence was found in a different fMRI study that demonstrated heightened activity in the right aIC during the retrieval of recently learned face-name pairs (Caviezel et al., 2020). In addition, these activation patterns were contrasted by prominent deactivation clusters in the PMR, suggesting an additional inhibitory function of the aIC on non-task-related memory networks (Caviezel et al., 2020; Ghahremani et al., 2015).

The preceding literature supports the functional dissociation of episodic and semantic memory retrieval. More recent literature, however, raises the question to what extent these memory processes can be separated (Nielson et al., 2010; Vatansever et al., 2021). Previous neuroimaging studies that have explored AM processes using faces of famous compared to non-famous individuals employed varied tasks with demands ranging from passive viewing to matching faces and fame discrimination (Nielson et al., 2010). Within this context, the most prominent regions for common activation included the HPC, fusiform and lingual gyrus, temporal cortices, and notably, the vmPFC, PCC, precuneus, and ATL (Nielson et al., 2010).

The current study builds upon a prior investigation by Caviezel et al. (2020), which explored the neural mechanisms of encoding and retrieval in AM. The goal was to add to their findings and enhance our comprehension of the neurofunctional correlates underlying recent and remote AM retrieval, a process that still needs further elucidation. For this purpose, we operationalised an associative retrieval task, where subjects had to validate recently learned face-name pairs (recent memory) and face-name pairs of famous people (remote memory). We adopted for the encoding the contingency learning procedure used by Caviezel et al. (2020), which was supposed to induce associative learning. This novel approach allowed us to study the neural correlates of explicit face-name retrieval within a contingency-based learning paradigm.

## 2 Methods

### 2.1 Design

The present study's primary emphasis was to directly assess the neurofunctional anatomy of the recent and remote memory systems using paired AM-related face-name representations. With this intent in mind, an associative retrieval task was implemented in which subjects were required to validate between two sets of distinct face-name pairs. The first set comprised recently learned face-name pairs (rlf), representing the recent memory condition. The second set consisted of face-name pairs specifically of famous people (ff), defining the remote memory condition. Additionally, we established baseline conditions for each experimental condition, where the participants were asked to verify the correct gender of the presented face. This was intentionally done to control for possible differences in sensory, cognitive, and motor demands. By contrasting the recent and remote memory conditions with their respective baselines, we aimed to isolate and examine the underlying neurofunctional signals associated with recent and remote AM retrieval processes. The local ethics committee approved the study design – Ethikkommission Nordwest- und Zentralschweiz (EKNZ) – which was conducted in accordance with the Declaration of Helsinki.

### 2.2 Participants

In total, 23 young, healthy individuals participated in the present study. The age range of our sample was between 19 and 31 years of age, with an average age of 23.39 years (*sd* = 3.23 years). Out of the participants, 7 were female, 21 were right-handed, and their average years of schooling were 13.74 (*sd* = 3.12 years). All participants were native German speakers with normal or corrected-to-normal vision and sufficient hearing abilities. They reported no personal history or family history of psychiatric disorders in first-degree relatives. Aside from a thorough neuropsychological and psychiatric screening, participants were given a mock task prior to scanning, in which they had to recognize the faces of various famous individuals. This was done to ensure that the participants were able to recognize famous individuals. Prior to their participation, all participants provided written informed consent. They were either compensated with university course credits (participation hours) or received a financial incentive for their participation.

### 2.3 Power analysis

To ensure the validity of the present study, a *post-hoc* power analysis was conducted using G^*^Power. The power was computed using an alpha error of 0.05 (two-sided), the sample size (N = 23), and a Cohen's d of 0.84, calculated by comparing the retrieval-related performance accuracy of the recent and remote memory tasks. The resulting power (1–β) of 0.97 can be interpreted as sufficient.

### 2.4 Task

During encoding, participants were presented with emotionally neutral faces with different names without prior instructions to memorize the face-name representations. Instead, they were given a mock task that involved rating the subjective fit of the presented face-name combinations. Six of the 12 faces were consistently paired with specific spoken names, forming a contingency condition to induce associative learning. Each face presented during the contingency condition was shown 12 times. Conversely, the remaining six faces were presented 12 times, each with a different name. This procedure served as a memory-free control condition, during which subjects could not form lasting associative memory representations. Accordingly, each face-name combination was presented once without repetition, thus avoiding any associative contingency learning while simultaneously controlling for the possible confounding effects of memory-independent sensory, cognitive, and motor demands.

**Figure 1:**
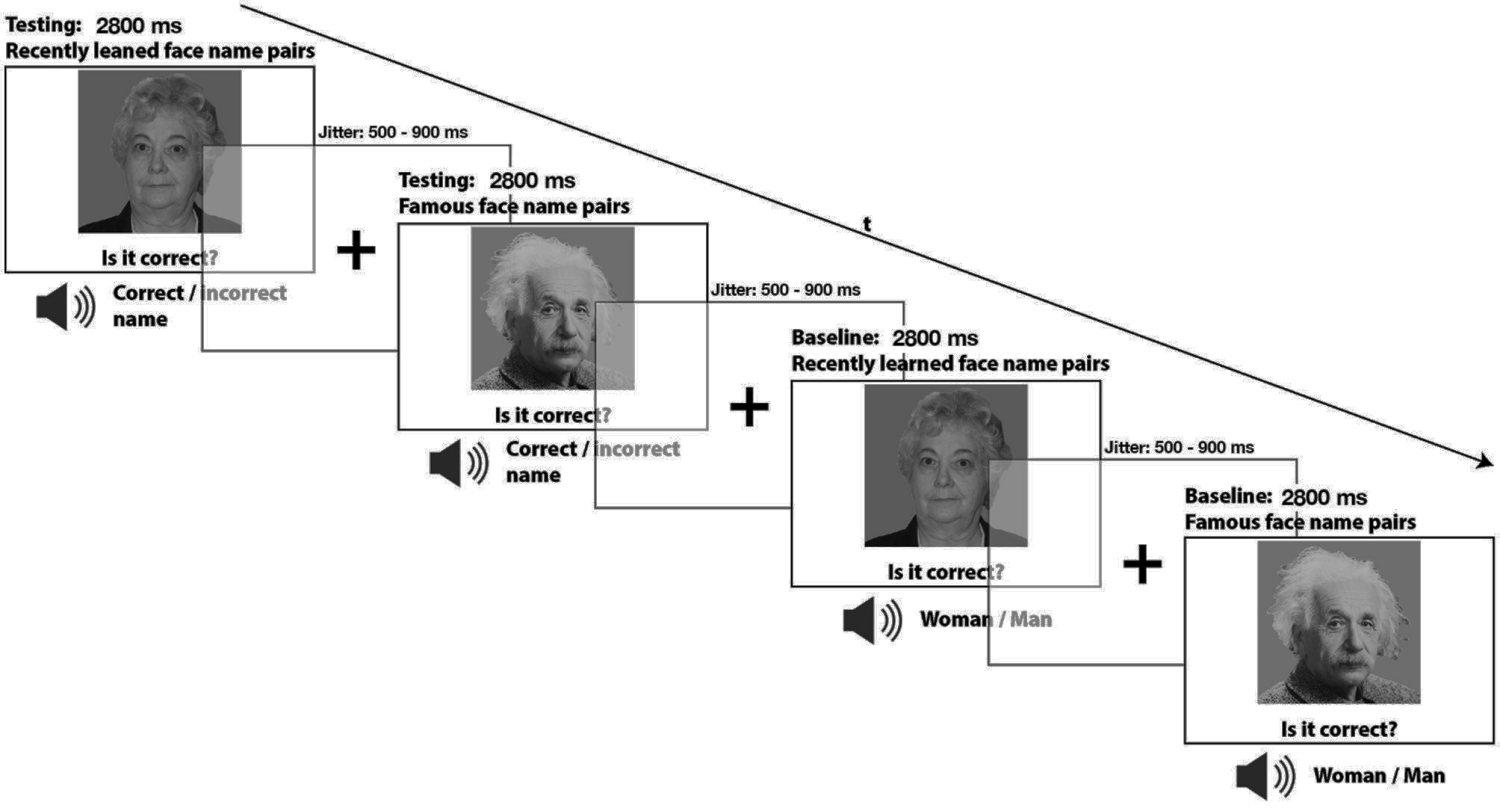
During the incidental retrieval task, participants were asked to recall the recently learned face-name pairs as well as the face-name combinations of famous people. A gender-matching task was also included as a baseline condition during the retrieval task, where participants only needed to determine if the presented image was of a male or female.

During the subsequent retrieval task, of which the test subjects were previously unaware, face-name pairs from the contingency condition representing the recent memory condition and (not previously learned) famous face-name pairs representing the remote memory condition were presented. These faces were not only associated with their original names but also with other (“incorrect”) names and required the participants to evaluate between a correct and incorrect face-name combination. As a result, the established contingent combinations were now disrupted, and participants had to consciously recall whether the current face-name combination was accurate or not. This explicit memory retrieval served as the main task. To establish suitable baseline conditions that would help identify brain activations related to retrieval processes, we presented participants with either the same recently learned faces used during encoding or with remotely learned faces of famous people. More specifically, both recently learned and famous faces were used, resulting in two baseline conditions. Each depiction of either face type was accompanied by the words “man” or “woman” using an artificially generated neutral male voice. These words replaced the name words used in the memory retrieval condition. In these matching tasks, participants had to determine whether the spoken word, which in this case represented gender labeling, corresponded to the depicted face. These two baseline conditions did not involve memory retrieval and were induced to provide appropriate baseline conditions that effectively controlled for non-mnemonic factors such as perception, motor demands and other cognitive processes (see Figure 1).

Every face-name pair (*n* = 6) of each retrieval category (recent vs. remote and correct vs. incorrect) was randomly presented six times, with intermittent presentation of every face-gender pair (*n* = 6) three times from the baseline category (recent vs. remote and correct vs. incorrect). This procedure led to a total of 216 events with 8 analysis conditions, of which the following are: recent memory correct face-name pairs (*n* = 36) and incorrect face-name pairs (*n* = 36), remote memory correct face-name pairs (*n* = 36) and incorrect face-name pairs (*n* = 36), baseline recent memory correct face-gender pairs (*n* = 18) and incorrect face-gender pairs (*n* = 18), baseline remote memory correct face-gender pairs (*n* = 18) and incorrect face-gender pairs (*n* = 18). All trial sequences were implanted in randomized order and counterbalanced for n-1 trial transitions and response side, i.e., left or right button press.

### 2.5 Stimuli

For the initial encoding and the subsequent recent memory retrieval task, 12 different human faces with emotionally neutral facial expressions were used. These images were carefully balanced for gender and age. Accordingly, half of the images (*n* = 6) depicted younger individuals, three males and three females, with a mean age of 19.83 years (sd = 1.17), while the other half (*n* = 6) depicted older individuals, three males and three females, with a mean age of 67.00 years (sd = 2.5). Both the recent and famous memory faces were presented during the entire task duration, each lasting for 2,800 ms. A button press did not interrupt the task. Jittered inter-stimulus intervals between 500 and 900 ms between stimuli were introduced, accompanied by a centered fixation cross. This was done to improve event separation and the hemodynamic response function estimation. All face stimuli were presented in black and white at a 22° angle using a mirror system implanted into the MR head coil and a projector screen placed at the foot end of the Scanner.

The names and words “man” or “woman”, simultaneously presented during the memory retrieval conditions and the gender-matching task, were presented acoustically to the test subjects and spoken by an artificial male voice. Each name lasted, depending on its length, i.e., “Elisabeth” or “man/woman”, approximately for 350 ms and 550 ms after the task had started. The participants received the auditory stimuli through two-sided stereo headphones. According to the Federal Statistics Office, the names used during the experiment were selected from the most commonly encountered names in the German-speaking parts of Switzerland (http://www.bfs.admin.ch). We also verified that the famous faces (e.g., Albert Einstein) and their respective first names (e.g., Albert) were well-known and culturally suitable and recognizable by young individuals at a university in Central Europe. The task and stimulus presentation described above were implemented using the E-Prime software (Version 2.0, Psychology Software Tools, Pittsburgh, PA, USA). Throughout the experiment, participants interacted using a push-button panel with two buttons and received the auditory stimuli through two-sided stereo headphones.

### 2.6 Image acquisition

Image acquisition was made using a 3T whole-body scanner (Magnetom Prisma, Siemens Healthcare, Erlangen, Germany) equipped with a 20-channel receiver head coil. For the T1 structural data, a three-dimensional (3D) magnetization-prepared rapid gradient-echo (MP-RAGE) pulse sequence (Mugler III and Brookeman, 1990) was used. T1 images had an isotropic spatial resolution of 1 mm, consisting of 176 sagittal slices (time of repetition (TR) = 2000 ms, echo time (TE) = 3.37 ms, flip angle (FA) = 8°, and field of view (FOV) = 256 × 256 × 176 mm3).

For the functional T2 data, a T2^*^-weighted, blood-oxygen-level-dependent (BOLD) sensitive interleaved gradient-echo planar imaging (EPI) sequence was utilized. All functional images had a spatial resolution of 3 × 3 mm^2^ and consisted of 39 transversal slices with a slice thickness of 3.0 mm (TR = 2,500 ms, TE = 30 ms, FA = 82°, and FOV = 228 × 228 mm^2^).

### 2.7 Behavioral data analysis

The behavioral performance data during retrieval encompassed the relative response accuracy, i.e., the percentage of correctly retrieved contingent or rejected non-contingent face-name pairs and the averaged response times (RT) for each condition. One-sample *t*-tests were utilized to test for differences in relative accuracy and reaction times between conditions. For statistical analyses, we employed the software package R Studio, R version 4.3.2 (2023-10-31).

### 2.8 fMRI data analysis

#### 2.8.1 Preprocessing and GLM specification

The data analysis was carried out using the SPM12 software (http://www.fil.ion.ucl.ac.uk/spm/). All EPI images, i.e., volumes, were realigned to the first volume for motion correction, coregistered to the anatomical volume for spatial alignment, normalized to the MNI305 T1 template, and lastly, spatially smoothed using a Gaussian kernel with a Full-Width at Half Maximum (FWHM) of 6 mm. During first-level GLM specification, the onset times for each trial of the experimental conditions were convolved with a canonical hemodynamic response function (HPR) to fit our intended event-related design. Serial correlations were removed with a first-order autoregressive model. Additionally, a high-pass filter with a cut-off of 128 seconds was used to remove low-frequency noise in the data. The six movement parameters derived from motion correction were included as nuisance covariates to account for any residual effects of head motion. All participants showed no translation higher than 2mm and no rotation greater than 2° in any direction. To test the hypothesis, the trials of all successfully retrieved face-name combinations during the recent and remote memory conditions were contrasted against their respective baseline trials (i.e., gender matching).

#### 2.8.2 Second level analyses

The resulting t-contrast images at the first-level analysis were then carried forward to the second-level analysis. At the second level, one sample t-tests, including conjunction analyses following the minimum statistic method (null conjunction), was performed to test for commonalities and differences in neural activations between recent and remote memory following whole-brain correction. The peak-voxel-level intensity threshold was set at a *p*-value of 0.05 and was corrected for Family-Wise-Error (FWE). In addition to the primary analyses using FWE-corrected thresholds (*p* < 0.05), we also reviewed and reported selected results of interest at uncorrected thresholds of *p* < 0.001 and *p* < 0.005 for exploratory and complementary purposes, which are included in the result section. The cytoarchitectonic atlas from the JuBrain Anatomy Toolbox, i.e., SPM Anatomy Toolbox (Eickhoff et al., 2005, 2006, 2007), was used to identify the relevant clusters. XJView (https://www.alivelearn.net/xjview) and MRIcroGL (https://www.nitrc.org/projects/mricrogl) were used for results visualization.

## 3 Results

### 3.1 Behavioral results

#### 3.1.1 Differences between conditions: accuracy

**Figure 2:**
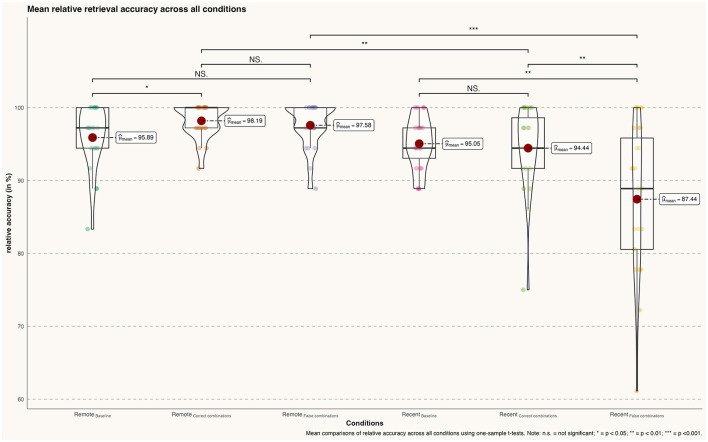
Differences in mean retrieval accuracy (percentage) across all conditions; n.s., not significant; *p < 0.05; **p < 0.01; ***p < 0.001.

Overall, participants successfully retrieved both recently learned and famous faces with a high degree of accuracy during all conditions. Nevertheless, significant differences between conditions were found. By comparison, participants' accuracy was significantly higher for famous faces' correct combinations than the baseline condition [Baseline (ff): *m* = 95.894 %, *sd* = 4.341; correct combinations (ff): *m* = 98.188 %, *sd* = 2.31; *t*_(22)_ = −2.238, *p* = 0.032, *d* = 0.66] but not when comparing the baseline with famous faces' false combinations [Baseline (ff): *m* = 95.894, *sd* = 4.341 %; false combinations (ff): *m* = 97.585 %, *sd* = 3.055; *t*_(22)_ = −1.528, *p* = 0.135, *d* = 0.45]. Comparing the relative accuracy of correct and false combinations during remote memory retrieval did not reveal a significant effect [correct combinations (ff): *m* = 98.188 %, *sd* = 2.31; false combinations (ff): *m* = 97.585 %, *sd* = 3.055; correct combinations (ff) *t*_(22)_ = 0.756, *p* = 0.454, *d* = 0.22] (see Figure 2).

Similar to the remote memory conditions, participants demonstrated a high degree of accuracy during recent memory conditions. Although the comparison between recent memory baseline and correct combinations of recently learned faces was not significant [Baseline (rlf): *m* = 95.048 %, *sd* = 4.00; correct combinations (rlf): *m* = 94.44 %, *sd* = 6.00; *t*_(22)_ = 0.423, *p* = 0.675, *d* = 0.12], the baseline comparison with false combinations did reach significance [Baseline (rlf): *m* = 95.048 %, *sd* = 4.00; false combinations (rlf): *m* = 87.440 %, *sd* = 10.00; *t*_(22)_ = 3.340, *p* = 0.002, *d* = 1.00]. In addition, the direct comparison between correct and false combinations of recently learned faces revealed a significant effect [correct combinations (rlf): *m* = 94.445, *sd* = 6.00; false combinations (rlf): *m* = 89.440, *sd* = 10.00; *t*_(22)_ = 2.886, *p* = 0.007*, d* = 0.85].

Lastly, participants achieved significantly higher accuracy in retrieving famous face-name pairs compared to recently learned face-name pairs during both correct [correct combinations (ff): m = 98.188, sd = 2.31; correct combinations (rlf): *m* = 94.44, *sd* = 6.00;*t*_(22)_ = 2.850, *p* = 0.008, *d* = 0.84] and false face-name combinations [false combinations (ff): *m* = 97.585, *sd* = 3.055; false combinations (rlf): *m* = 87.440, *sd* = 10.00; *t*_(22)_ = 4.59, *p* < 0.001, *d* = 1.35].

#### 3.1.2 Differences between conditions: reaction times

**Figure 3:**
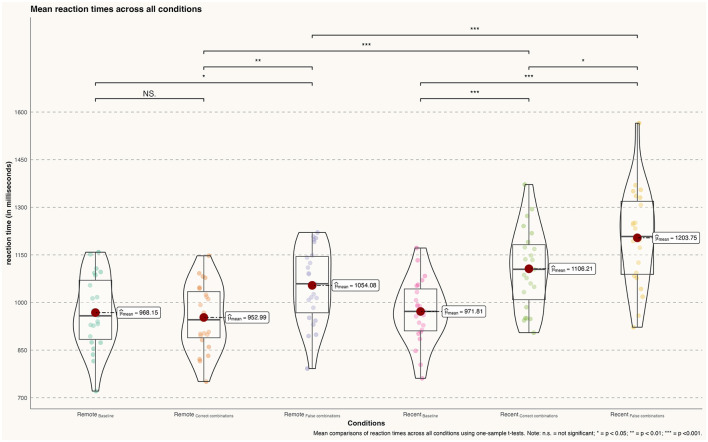
Differences in mean reaction times (in milliseconds) across all conditions; n.s., not significant; *p < 0.05; **p < 0.01; ***p < 0.001.

During the retrieval conditions of famous faces, participants did not show significantly increased reaction times when comparing the famous faces baseline and correct combinations [Baseline (ff): *m* = 0.968 ms, *sd* = 0.115; correct combinations (ff): *m* = 0.953 ms, *sd* = 0.104; *t*_(22)_ = 0.468, *p* = 0.642, *d* = 0.14]. However, a significant effect was found when comparing the baseline with famous faces false combinations [Baseline (ff): *m* = 0. 68 ms, *sd* = 0.115; false combinations (ff): *m* = 1.054 ms, *sd* = 0.118; *t*_(22)_ = −2.499, *p* = 0.016, *d* = 0.74]. The direct comparison between correct face–name combinations and incorrect combinations of famous faces revealed that participants exhibited a significantly increased reaction time during the false combinations condition compared to the correct combinations condition [correct combinations (ff): *m* = 0.953 ms, *sd* = 0.104; false combinations (ff): *m* = 1.054 ms, *sd* = 0.118; *t*_(22)_ = −3.074, *p* = 0.004, *d* = 0.91] (see Figure 3).

During recent memory conditions, participants showed significantly increased reaction times when retrieving correct [Baseline (rlf): *m* = 0.972 ms, *sd* = 0.10; correct combinations (rlf): *m* = 1.106 ms, *sd* = 0.126; *t*_(22)_ = −4.00, *p* < 0.001, *d* = 1.18] and false combinations [Baseline (rlf): *m* = 0.972 ms, *sd* = 0.10; false combinations (rlf): *m* = 1.204 ms, *sd* = 0.151; *t*_(22)_ = 6.140, *p* < 0.001, *d* = 1.81] of recently learned face–name pairs compared to the baseline condition. In addition, participants demonstrated slower rt during the retrieval of false combinations of face–name pairs compared to correct combinations [correct combinations (rlf): *m* = 1.106 ms, *sd* = 0.126; false combinations (rlf): *m* = 1.204 ms, *sd* = 0.151; *t*_(22)_ = −2.376, *p* = 0.02, *d* = 0.70].

Analogous to the accuracy, participants' reaction times were significantly increased during the retrieval of recently learned face–name pairs compared to remotely learned pairs both for correct combinations [correct combinations (ff): *m* = 0.953 ms, *sd* = 0.104; correct combinations (rlf): *m* = 1.106 ms, *sd* = 0.126; *t*_(22)_ = −4.487, *p* < 0.001, *d* = 1.32] as well as [false combinations (ff): *m* = 1.054 ms, *sd* = 0.118; false combinations (rlf): *m* = 1.204 ms, *sd* = 0.151; *t*_(22)_ = −3.740, *p* < 0.001, d = 1.10].

### 3.2 fMRI results

During recent memory retrieval (recent memory > baseline), activation was observed bilaterally in the aIC and in the ATL, mainly comprising the anterior divisions of the STG. Notably, regions exhibiting deactivation for recent memory were particularly prominent. The primary cluster demonstrating bilateral deactivation, included the PCC - on the border with the central sulcus- and the medial area of the precuneus as components of the PMR. Additionally, main clusters of deactivations (baseline > recent memory) were evident in the bilateral MTG, encompassing the inferior temporal gyrus, the right lingual gyrus, the bilateral lateral occipital cortex, the right superior parietal lobe, the left occipital pole, the left supramarginal gyrus, and the right postcentral gyrus (see Tables 1, 2).

**Table 1:** Significant activations during recent and remote memory retrieval.

		**Recent activation**					**Remote activation**					**Conjunction**				
**Region**	**H**	**X**	**Y**	**Z**	**Pseudo** ***t*****-value**	**k**	**X**	**Y**	**Z**	**Pseudo** ***t*****-value**	**k**	**X**	**Y**	**Z**	**Pseudo** ***t*****-value**	**K**
*Superior temporal gyrus, ant. division (ATL)*	*L*	*−56*	*−12*	*2*	*7.74*	*102*	*−60*	*−6*	*-4*	*7.96*	*195*	*-58*	*-10*	*0*	*7.63*	*60*
*Superior temporal gyrus, ant. division (ATL)*	*R*	*62*	*−4*	*0*	*5.12*	*1*	*64*	*−6*	*-2*	*5.67*	*23*	*62*	*-6*	*0*	*4.93*	60^*^
*Anterior insular cortex*	*R*	*32*	*24*	*−6*	*5.61*	*15*	*N. S*.					*N. S*.				
*Anterior insular cortex*	*L*	*−30*	*22*	*−4*	*5.3*	*1*	*N. S*.					*N. S*.				
*Precuneus (PMR)*	*M*	*N. S*.					*0*	*−60*	*24*	*5.94*	*37*	*N. S*.				
*Posterior cingulate cortex (PMR)*	*L*	*N. S*.					*−4*	*−52*	*18*	*5.47*	*10*	*N. S*.				
*Ventromedial prefrontal cortex*	*L*	*N. S*.					*−8*	*30*	*−12*	*5.38*	*1*	*N. S*.				

Using the Statistical nonparametric Mapping (SnPM) toolbox, pseudo t-values have been obtained after variance smoothing. The reported values are thresholded at p_FWE_ < 0.05 at peak level (^*^uncorrected P < 0.001 at peak level); H, hemisphere; L, left; R, right; M, medial; k, cluster size; N.S., not significant; coordinates are in MNI space; ATL, anterior temporal lobe; PMR, posterior midline region.

**Table 2:** Significant deactivations for remote and recent memory.

		**Recent deactivation**					**Remote deactivation**					**Conjunction**				
**Region**	**H**	**X**	**Y**	**Z**	**Pseudo** ***t*****-value**	**k**	**X**	**Y**	**Z**	**Pseudo** ***t*****-value**	**k**	**X**	**Y**	**Z**	**Pseudo** ***t*****-value**	***k***
*Supramarginal gyrus*	*L*	*−58*	*−44*	*38*	*5.71*	*37*	*−56*	*−42*	*44*	*5.31*	*5*	*−56*	*−42*	*44*	*5.07*	*3*
*Postcentral gyrus*	*R*	*36*	*−30*	*36*	*5.53*	*10*	*36*	*−32*	*40*	*5.13*	*1*	*36*	*−32*	*38*	*4.61*	117^*^
*Posterior cingulate cortex (PMR)*	*L*	*−2*	*−20*	*44*	*7.83*	*704*	*N. S*.					*N. S*.				
*Middle temporal gyrus, temporooccipital part*	*L*	*−60*	*−46*	*0*	*7.27*	*1,084*	*N. S*.					*N. S*.				
*Lingual gyrus*	*R*	*30*	*−44*	*−8*	*7.12*	*98*	*N. S*.					*N. S*.				
*Inferior temporal gyrus, temporooccipital part*	*R*	*58*	*−56*	*−12*	*6.83*	*357*	*N. S*.					*N. S*.				
*Middle temporal gyrus, temporooccipital part*	*R*	*62*	*−42*	*0*	*6.39*	–	*N. S*.					*N. S*.				
*Lingual gyrus*	*R*	*14*	*−70*	*−12*	*6.46*	*62*	*N. S*.					*N. S*.				
*Lateral occipital cortex*	*R*	*42*	*−76*	*8*	*6.33*	*377*	*N. S*.					*N. S*.				
*Lateral occipital cortex*	*L*	*−38*	*−82*	*22*	*6.11*	*38*	*N. S*.					*N. S*.				
*Superior parietal lobule*	*R*	*42*	*−40*	*62*	*6.05*	*18*	*N. S*.					*N. S*.				
*Occipital pole*	*L*	*−12*	*−92*	*24*	*5.96*	*80*	*N. S*.					*N. S*.				

Using the Statistical nonparametric Mapping (SnPM) toolbox, pseudo t-values have been obtained after variance smoothing. The reported values are thresholded at p_FWE_ < 0.05 at peak level (^*^uncorrected P < 0.001 at peak level); H, hemisphere; L, left; R, right; M, medial; k, cluster size; N.S., not significant; coordinates are in MNI space; – = subcluster.

During famous face-name pair retrieval (remote memory > baseline), fMRI results showed bilateral activation clusters in the ATL -again comprising mostly the anterior divisions of the STG and marginally the MTG- and the precuneus, near the posterior subparietal sulcus, along with unilateral left sided clusters of the ventral PCC and the vmPFC. The PCC and precuneus activation clusters in the PMR did not correspond with deactivation clusters found during the recent memory condition. Deactivations (baseline > remote memory) were observed in the left supramarginal gyrus and the right postcentral gyrus.

**Figure 4:**
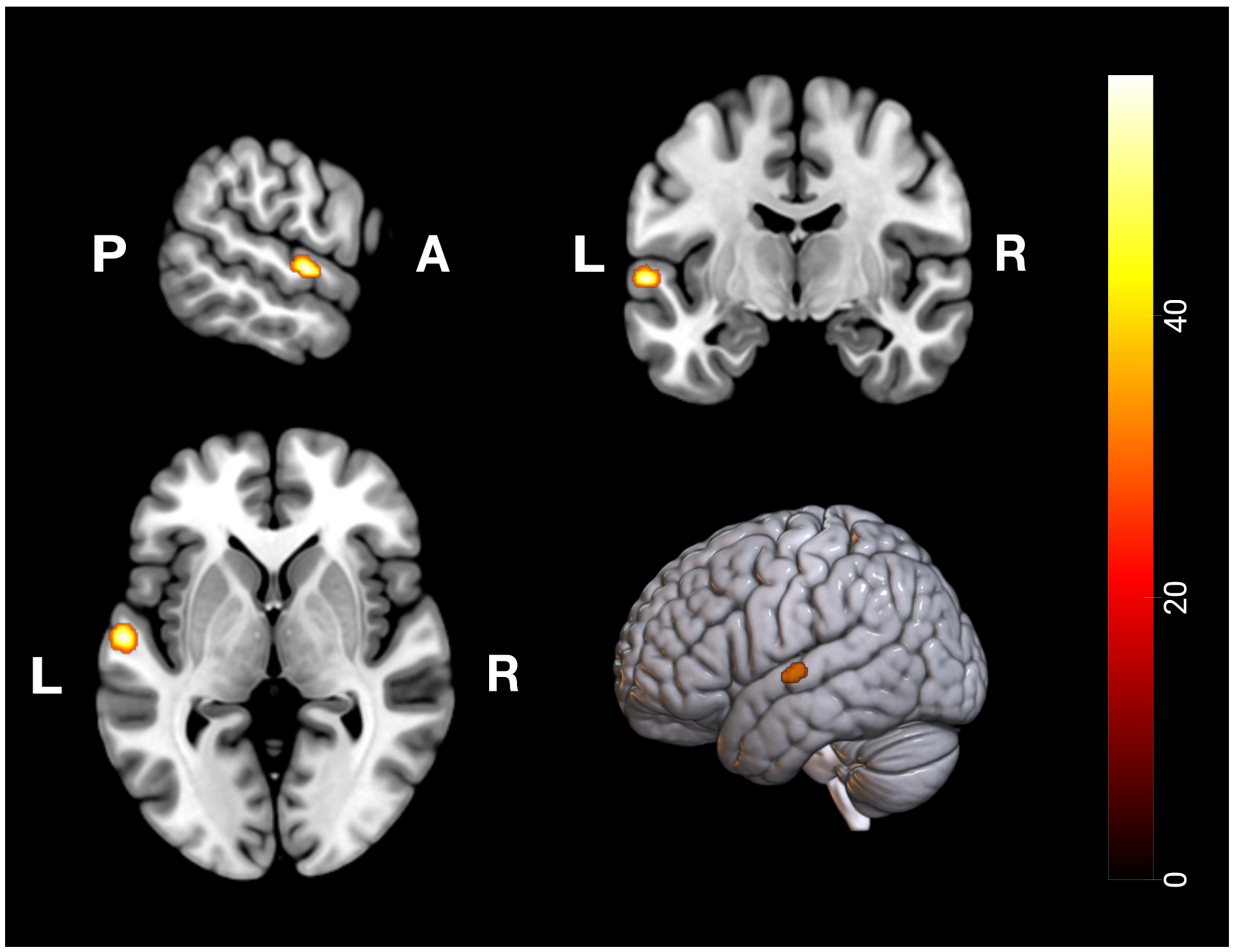
Conjunction Analysis: Depiction of common neuronal activity in the anterior division of the left superior temporal gyrus as part of the ATL during the remote and recent memory condition; Activation thresholded at *p* < 0.05, FWE corrected.

Conjunction analysis of recent and remote memory retrieval (recent memory > baseline n remote memory > baseline) revealed significant overlap in activation clusters in the left ATL, comprising the anterior division of the STG, following FWE correction (see Figure 4). Common deactivations (baseline > recent memory n baseline > remote memory) occurred in the left anterior division of the supramarginal gyrus and the right postcentral gyrus (see Tables 1, 2).

The direct comparison of remote vs. recent memory retrieval with baseline ([remote memory > baseline] > [recent memory > baseline]) revealed significant bilateral activation in the temporo-occipital parts of the middle temporal gyrus and the lateral occipital cortex. Further analysis at an uncorrected *p* < 0.001 threshold revealed significant activation clusters in the PCC, the precuneus, and the vmPFC, which correspond to activation clusters observed during remote memory retrieval. For the direct comparison without baseline (remote memory>recent memory) the activation clusters in the PCC, precuneus and vmPFC were also observed at FWE-corrected threshold (p < 0.05) (see Table 3).

**Table 3:** Significant activations during remote vs recent memory retrieval.

	**Direct comparison with baseline**						**Direct comparison without baseline**				
***Region***	***H***	***X***	***Y***	***Z***	***Pseudo T-value***	***k***	***X***	***Y***	***Z***	***Pseudo T-value***	***k***
Middle temporal Gyrus, temporooccipital part	R	*66*	*−40*	*0*	*5.95*	*60*	*N. C*.				
Superior temporal Gyrus, posterior division	L	*−52*	*−14*	*−8*	*5.23*	*7*					
Lateral occipital cortex, inferior division	R	*52*	*−70*	*−6*	*5.22*	*6*	*52*	*−70*	*−2*	*6.83*	*131*
Middle temporal gyrus, temporooccipital part	L	*−58*	*−50*	*−10*	*5.12*	*3*	*−56*	*−50*	*−8*	*6.95*	*118*
Lateral occipital cortex, superior division	R	*32*	*−72*	*32*	*5.07*	*3*	*N. C*.				
Posterior cingulate cortex/Precuneus (PMR)	L	*−6*	*−52*	*4*	*4.57*	246^*^	*−6*	*−52*	*6*	*7.43*	*261*
Ventromedial prefrontal cortex	L	*−6*	*26*	*−10*	*4.4*	117^*^	*−2*	*24*	*−12*	*6.67*	*104*
Parahippocampus, posterior division	L	*−24*	*−30*	*−22*	*3.66*	105^*^	*−26*	*−32*	*−18*	*6.52*	*81*
Lateral occipital cortex, superior division	R	*54*	*−64*	*20*	*4.77*	−^*^	*42*	*−60*	*20*	*5.89*	*52*
Postcentral gyrus	R	*12*	*−38*	*54*	*4.04*	38^*^	*14*	*−40*	*56*	*6.22*	*32*
Posterior cingulate cortex	R	*8*	*−54*	*10*	*4.15*	33^*^	*6*	*−50*	*8*	*5.39*	*8*

Using the Statistical nonparametric Mapping (SnPM) toolbox, pseudo t-values have been obtained after variance smoothing. The reported values are thresholded at p_FWE_ < 0.05 and at uncorrected p < 0.001 (blue^*^) at peak level; for activations without baseline only thresholded results at p_FWE_ < 0.05 at peak level were included; H, hemisphere; L, left; R, right; M, medial; k, cluster size; coordinates are in MNI space; N.C., not corresponding; green highlighted regions correspond with the findings of the remote memory condition (remote memory >baseline); – = subcluster.

The direct comparison of recent vs. remote memory retrieval with baseline ([remote memory > baseline] > [recent memory > baseline]) revealed significant activation clusters at an uncorrected *p* < 0.001 level in the right paracingulate gyrus, bordering the superior frontal gyrus, and at an uncorrected *p* < 0.005 level, bilateral activation clusters in the aIC. The location of the cluster in the left aIC corresponds directly to the activation observed in the aIC during recent memory retrieval. For the direct comparison without baseline (recent memory > remote memory), bilateral activation clusters of the aIC were also observed at an FWE-corrected threshold (*p* < 0.05), both of which spatially overlapped with the aIC clusters observed during the recent memory condition (see Table 4).

**Table 4:** Significant activations during recent vs remote memory retrieval.

	**Direct comparison with baseline**						**Direct comparison without baseline**					
***Region***	***H***	***X***	***Y***	***Z***	***Pseudo T-Value***	***k***	***H***	***X***	***Y***	***Z***	***Pseudo T-value***	***k***
Paracingulate gyrus/Superior frontal gyrus	*R*	*8*	*16*	*50*	*3.8*	16^*^	*L/R*	*0*	*28*	*38*	*7.68*	*393*
Paracingulate gyrus/Superior frontal gyrus	*R*	*6*	*30*	*42*	*3.71*	7^*^	*L/R*	*0*	*28*	*38*	*7.68*	*393*
Anterior insular cortex	*L*	*−30*	*22*	*−6*	*3.61*	17^**^	*L*	*-32*	*18*	*2*	*6.31*	*100*
Paracingulate gyrus	*L*	*−6*	*24*	*44*	*3.06*	14^**^	*L/R*	*0*	*28*	*38*	*7.68*	*393*
Anterior insular cortex	*R*	*34*	*20*	*6*	*3.02*	2^**^	*R*	*34*	*20*	*4*	*5.19*	*3*
Heschls. gyrus	*R*	*54*	*−14*	*2*	*2.03*	1^**^	*R*	*56*	*-12*	*4*	*6.02*	*22*
Anterior insular cortex	*N.C*.						*R*	*34*	*22*	*−8*	*6.32*	*70*
Heschls. gyrus							*L*	*−48*	*22*	*4*	*5.56*	*21*

Using the Statistical nonparametric Mapping (SnPM) toolbox, pseudo t-values have been obtained after variance smoothing. The reported values are thresholded at p_FWE_ < 0.05 and at uncorrected p < 0.001 (blue^*^) and uncorrected p < 0.005 (dark blue^**^) at peak level; for activations without baseline only thresholded results at p_FWE_ < 0.05 at peak level were included; H =, hemisphere; L, left; R, right; M, medial; k, cluster size; coordinates are in MNI space; N.C., not corresponding; green highlighted regions correspond with the findings of the recent memory condition (recent memory >baseline).

## 4 Discussion

**Figure 5:**
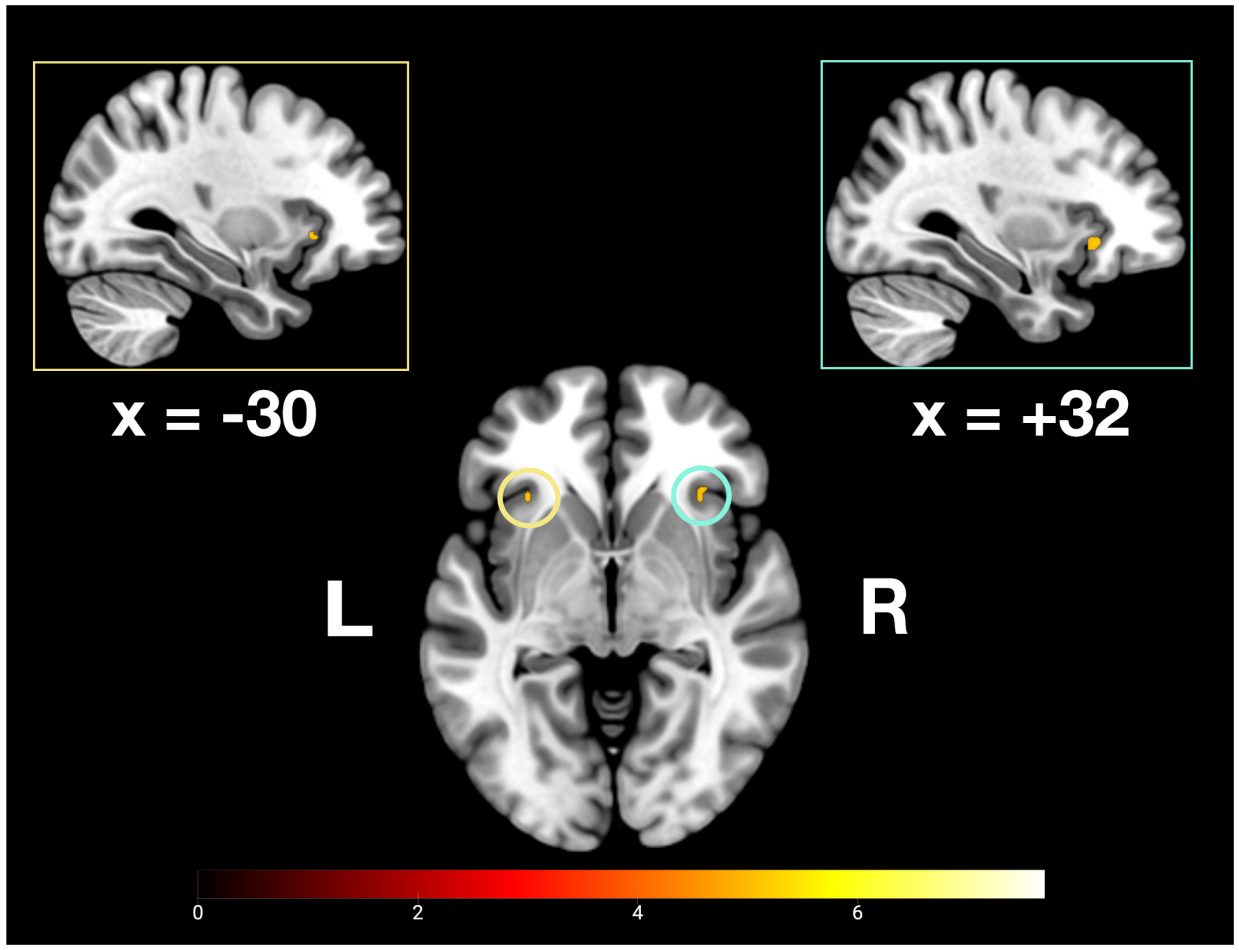
Depiction of bilateral neuronal activity in the anterior insular cortex during the recent memory condition; Activation thresholded at *p* < 0.05, FWE corrected.

The present study explored commonalities and distinctions among the neurofunctional correlates of recent and remote memory retrieval using associative face-name pairs as stimuli. We assumed to detect common and differing brain activity in semantic and remote memory processing regions. Building upon prior research by Caviezel et al. (2020), we also anticipated additional brain activity in areas prominently associated with higher cognitive control, such as prefrontal and parietal regions. The key findings of this study revealed bilateral activation in the anterior divisions of the STG as part of the ATL during remote and recent memory retrieval. During recent memory retrieval, specific activation included bilateral activation clusters in the aIC (see Figures 5, 9).

**Figure 6:**
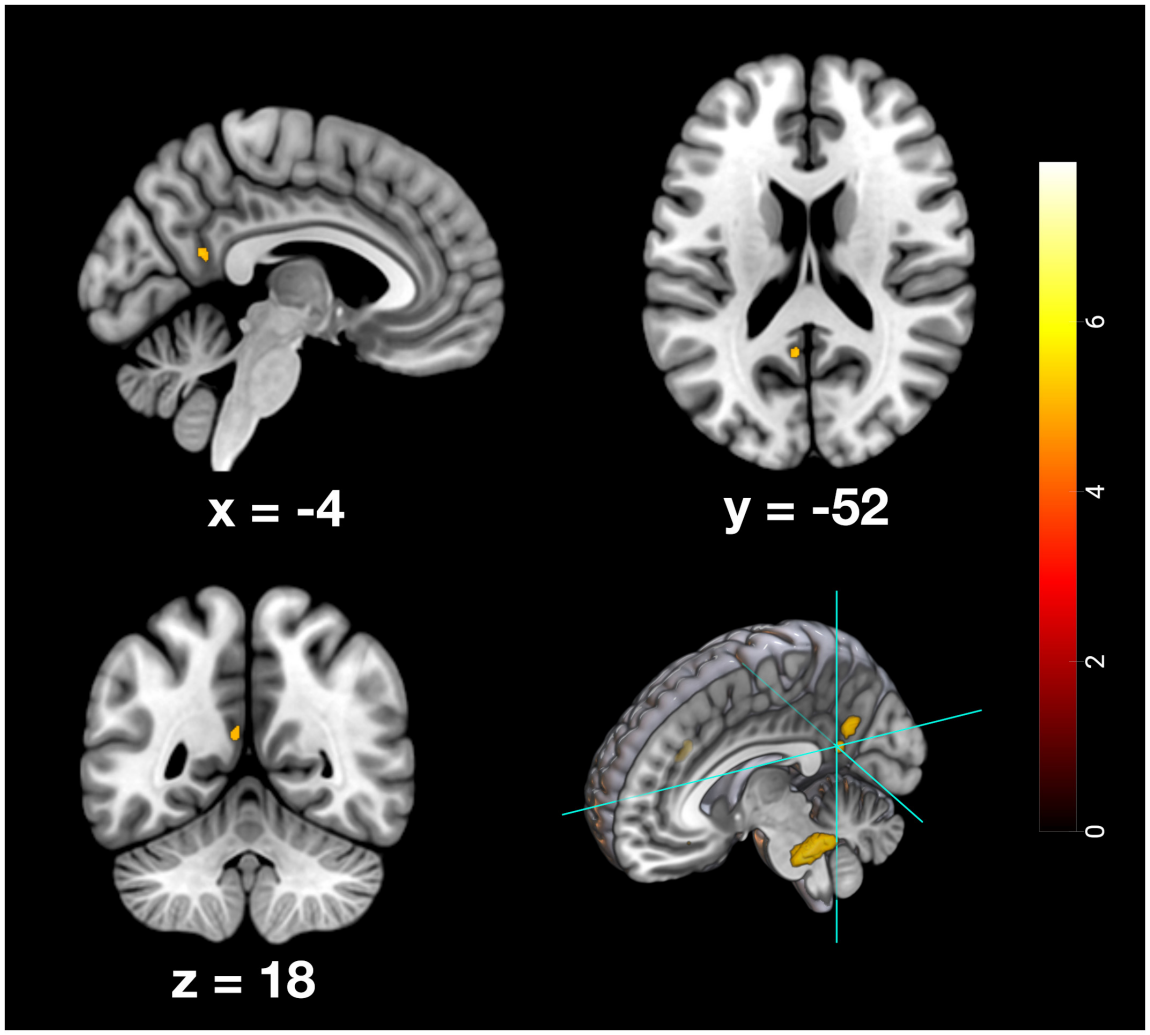
Depiction of neuronal activity in the posterior cingulate cortex as part of the PMR during the remote memory condition; Activation thresholded at *p* < 0.05, FWE corrected.

**Figure 7:**
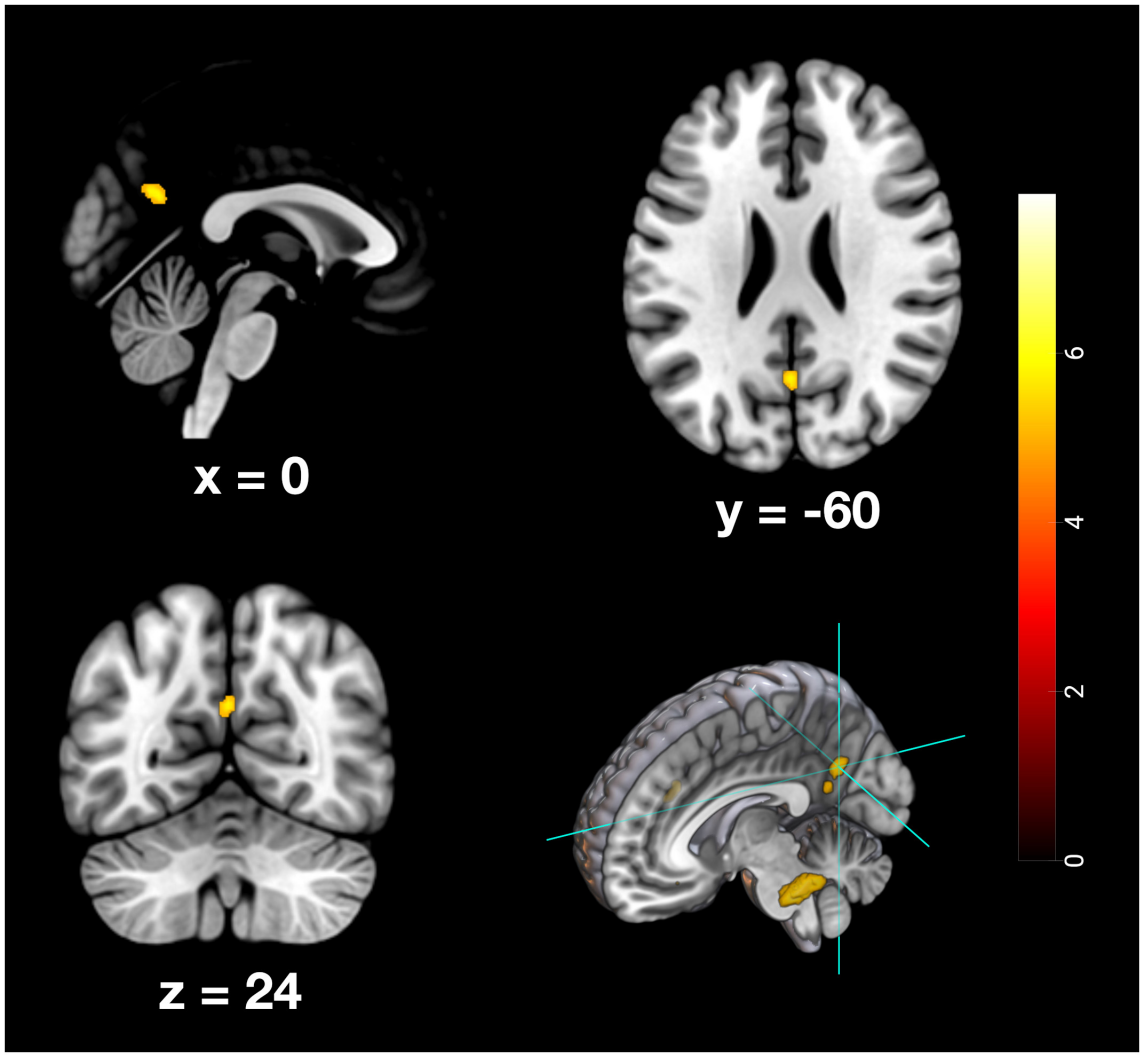
Depiction of neuronal activity in the Precuneus as part of the PMR during the remote memory condition; Activation thresholded at *p* < 0.05, FWE corrected.

**Figure 8:**
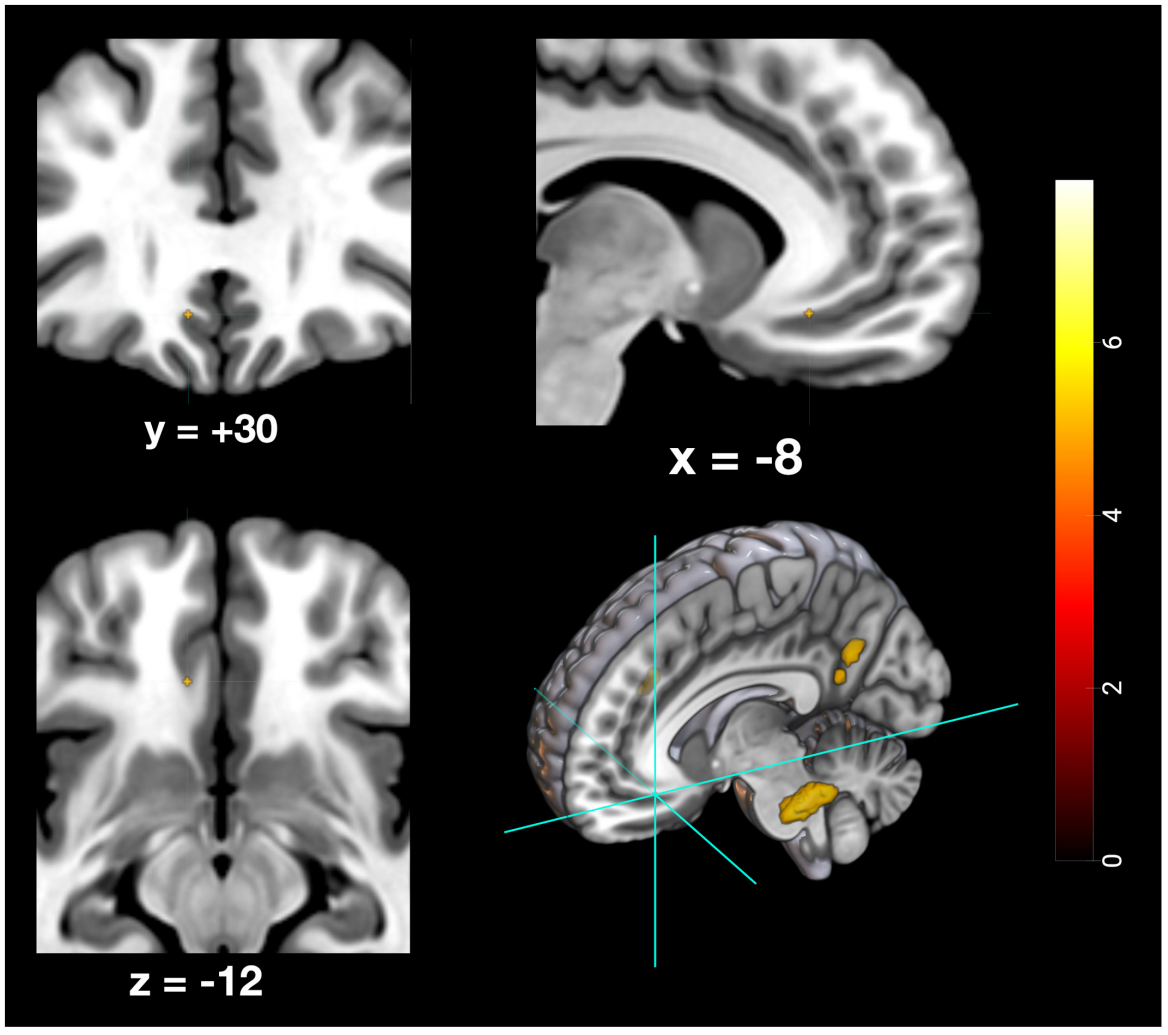
Depiction of neuronal activity in the ventromedial prefrontal cortex during the remote memory condition; Activation thresholded at *p* < 0.05, FWE corrected.

**Figure 9:**
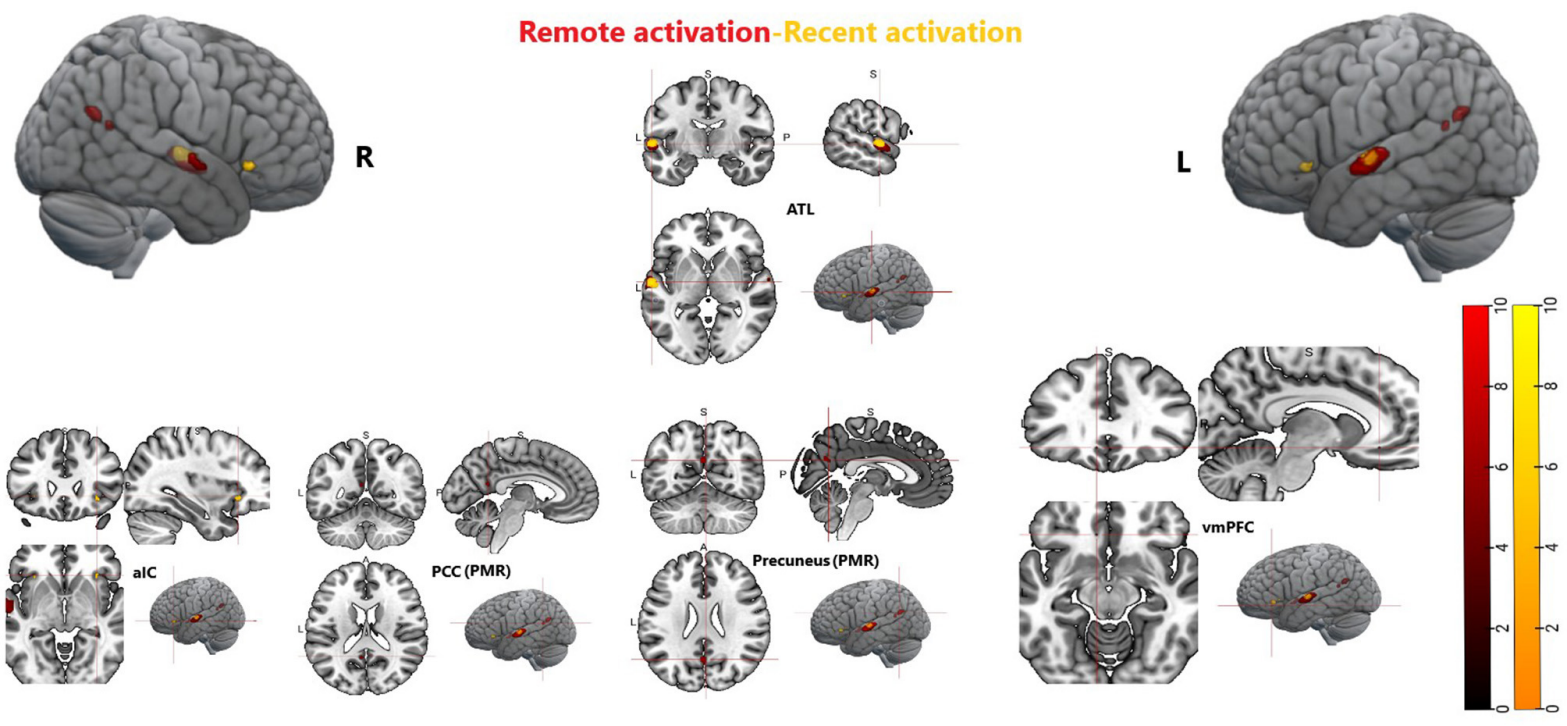
Whole brain activation during recent memory and remote memory, Activation thresholded at *p* < 0.05, FWE corrected. ATL, anterior temporal lobe; aIC, anterior insular cortex; PCC, posterior cingulate cortex; vmPFC, ventromedial prefrontal cortex; PMR, posterior midline region.

**Figure 10:**
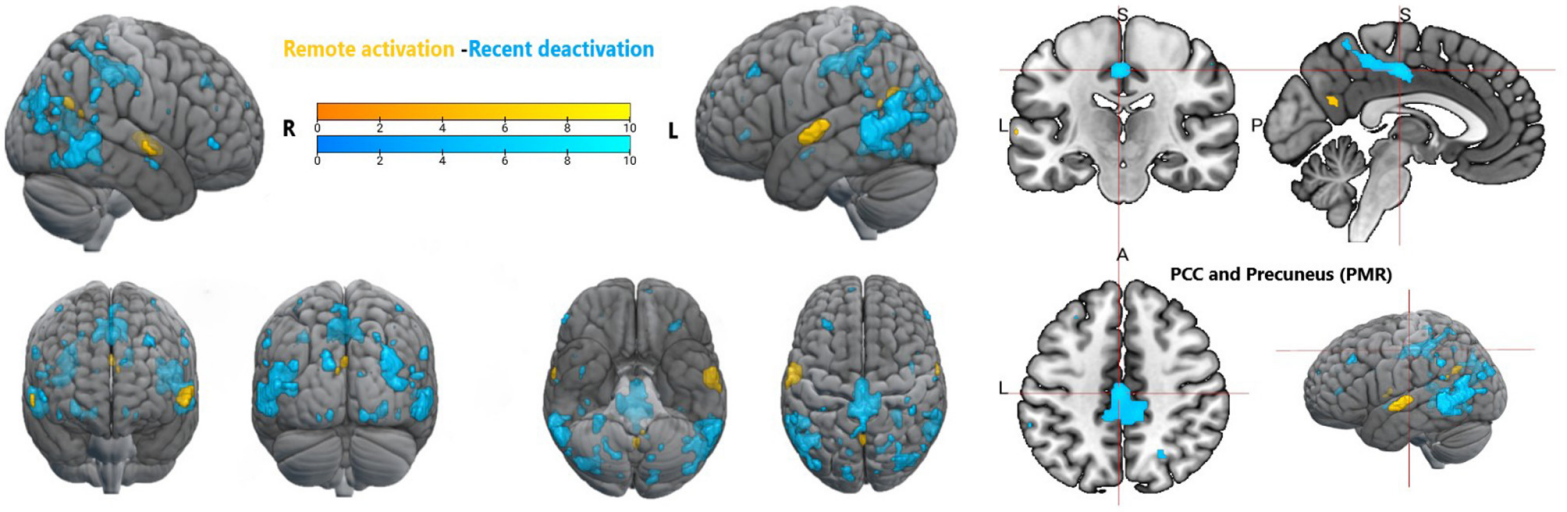
Whole brain activation during remote memory and deactivation during recent memory, Activation thresholded at *p* < 0.05, FWE corrected. PCC, posterior cingulate cortex; PMR, posterior midline region.

Furthermore, specific activation clusters were observed during remote memory retrieval in the vmPFC and the PMR, specifically encompassing the pMCC and precuneus (see Figures 6–9). In addition, common deactivations were found only in the supramarginal and postcentral gyrus, and prominent PMR deactivations were found during recent memory retrieval (see Figure 10). No distinct deactivations were observed for remote memory retrieval. In contrast to previous findings in recent and remote memory processing, no significant contributions of the HPC proper or the MTL at FWE level were observed in the present study. The findings will be discussed in more detail further below.

### 4.1 Recent and remote memory retrieval relies on the anterior STG as a key structure of the ATL

The bilateral activation clusters in the anterior divisions of the STG, as part of the ATL, observed during both recent and remote memory conditions, align with previous findings on semantic processing. However, the literature also points out that the likelihood of observing ATL activation depends on the imaging modality used (e.g., PET vs. fMRI), as well as the specificity of the semantic task (Patterson et al., 2007; Visser et al., 2010). In particular, auditory stimuli—such as words or sentences—are generally associated with stronger ATL activation compared to visually based tasks (Visser et al., 2010). The ATL as a whole is widely recognized as a key region involved in the retrieval of factual information about individuals (Nielson et al., 2010). However, the specific subregions of the ATL activated during a task can vary depending on the sensory modality of the stimulus (Visser et al., 2010). Whether the information is presented auditorily or visually influences not only whether more ventral vs. posterior or superior vs. inferior regions of the temporal lobe are engaged, but also which hemisphere of the brain is predominantly activated (Nielson et al., 2010; Visser et al., 2010). According to a meta-analysis by Visser et al. (2010), semantic tasks that combine pictures and auditory words often tend to engage superior regions of the ATL, such as the STG. Furthermore, the distinction between phonemic and non-phonemic sounds within a task also influences which regions of the STG are activated. fMRI trials based on phonemic sounds (i.e., those serving language) generally show stronger activation in ventral areas of the STG compared to non-phonemic trials (Humphries et al., 2014). Additionally, the duration of auditory stimuli can affect BOLD responses during tasks (Rinne et al., 2005). We did not control for sound duration in our study; however, due to the natural variability in first name length (~350 ms and 550 ms), we did not anticipate it would meaningfully impact our outcomes.

Considering the auditory and linguistic aspects of our semantic task, we expected the anterior divisions of the STG to be particularly active. One meta-analysis defined four subdivisions of the ATL, one of which is the inferior dorsal ATL, extending from the frontal pole to the anterior STG. This subdivision was particularly associated with auditory sounds, voice identity, sentence comprehension and processing, and, interestingly, also with word semantics, audiovisual modality, and memory retrieval (Hung et al., 2020). Notably, the audiovisual and memory-related aspects of this ATL subdivision correspond with our findings. During both remote and recent memory retrieval, our participants processed audiovisual stimuli and retrieved associative memories. The specific modality of our task—retrieving face-name pairs—also parallels findings from another study, which reported that the anterior superior temporal sulcus was particularly active during the audiovisual presentation of face-name associations (Lee et al., 2017). Overall, these findings from previous studies complement our results and suggest that the anterior portion of the superior temporal lobe functions as a hub for audiovisual integration and associative memory.

Adding another layer of evidence, our task component involving famous faces and names is also known to elicit prominent ATL activation (Nielson et al., 2010; Patterson et al., 2007). Consistent with this, the ATL clusters observed during remote memory retrieval were more pronounced than those during recent memory retrieval. In line with the evidence outlined above, we argue that the semantic specificity of our task favored the anterior divisions of the STG over other brain regions associated with semantic memory (e.g., the posterior inferior parietal lobe). Notably, the novelty of our findings lies in the observation that retrieval of associative memories for both remotely and recently learned face-name pairs appears to substantially rely on the anterior STG as a subregion of the ATL.

### 4.2 Recent memory retrieval involves the aIC and deactivates the PMR

In addition to bilateral ATL activity, we observed significant bilateral activation of the aIC during recent memory retrieval. Traditionally, the aIC is recognized as a central hub of the salience network, becoming active in response to salient stimuli—even those outside the current focus of attention (Muller et al., 2016; Seeley, 2019). Furthermore, the aIC has been identified as a neural switching mechanism mediating between task-positive networks, such as the central executive and dorsal attention networks, and task-negative networks, including the DMN, of which the PMR is a central hub (Wu et al., 2019).

The observed deactivation cluster in the PMR during recent memory retrieval suggests increased cognitive demands (Daselaar et al., 2009). Although this deactivation did not spatially overlap with the activation observed during the remote memory condition (see Results section), we interpret this as indicative of differing task demands, reflecting distinct neural correlates associated with recent memory retrieval. Notably, similar reversed activation patterns between the aIC and PMR have been observed in previous fMRI research, where the aIC was implicated in the inhibition of non-task-relevant brain regions (Caviezel et al., 2020). Moreover, the aIC has been implicated in the retrieval of both semantic and episodic memory, particularly when memory associations are weak (Vatansever et al., 2021). It has been argued that enhanced cognitive control is especially necessary during the retrieval of less consolidated -and therefore more effortful- associative memory representations (Engström et al., 2015; Vatansever et al., 2021).

Taken together, these findings support at least a partial contribution of the aIC to associative memory retrieval, particularly for recently acquired information. A separate study demonstrated that individual differences in cognitive control ability could be predicted from microstructural features of the aIC during a working memory task (dual n-back), further linking the aIC to the allocation of working memory resources (Menon, 2025; Menon et al., 2020). While most of the literature associates the aIC with (short-term) working memory facilitation (Menon, 2025; Vatansever et al., 2021) our findings suggest an active role of the aIC in (long-term) memory retrieval of recently acquired face-name pairs. However, systematic investigations into the specific role of the aIC in memory processes are still lacking. It remains to be determined whether its contribution lies solely in recruiting task-relevant memory networks, or whether the aIC is directly involved in the retrieval of memory representations.

### 4.3 Remote memory retrieval relies on semantic and episodic memory networks

The findings for our remote memory condition indicate that distant memories are more likely to comprise episodic and semantic characteristics and that retrieving such memories engages both types of memory networks. Accordingly, past brain imaging and clinical studies have consistently associated semantic memory retrieval with the ATL (Pisoni et al., 2020). Conversely, episodic memory retrieval has been associated primarily with the MTL and the precuneus, the PCC and the vmPFC (Gilboa and Moscovitch, 2021; Vatansever et al., 2021), which we observed specifically during remote memory retrieval. Both the vmPFC and the PMR are critical hubs of the DMN, which are active during the successful retrieval of distant memories, encompassing especially autobiographical memory information (Palacio and Cardenas, 2019; Sestieri et al., 2014). However, AM processes sometimes engage both semantic and episodic memory-related regions simultaneously during the same task (Nielson et al., 2010; Tanguay et al., 2022; Vatansever et al., 2021). This becomes even more apparent when participants associate and retrieve person-specific information, such as the faces and names of human individuals (Nielson et al., 2010). For instance, when retrieving remote face-name pairs, both memory networks might come into play, depending on the time and context in which the memory association was formed, and the specific content (birthdate, name or events) being retrieved. This notion finds support in an fMRI study conducted by Tanguay et al. (2022), which investigated the overlapping and separate regions involved in the retrieval of semantic and episodic memories and used four memory categories, which comprised the retrieval of general facts, autobiographical facts, repeated events, and unique events. Brain regions that exhibited bilateral activation across all four memory types included the precuneus, PCC, medial frontal cortex and, notably, the STG and MTG (Tanguay et al., 2022).

Recent literature suggests that the PMR and its constituent brain regions can be further divided into distinct functional subdivisions (Chen et al., 2017; Foster et al., 2023; Kwon et al., 2025). The PCC, for example, can be divided into dorsal and ventral areas, with the dorsal area more strongly associated with executive and decision-making functions, and the ventral area more closely linked to mnemonic processes, particularly autobiographical memory (Foster et al., 2023). Memory studies investigating the functional subdivisions of the PMR, namely the medial parietal cortex, an anatomical term that largely overlaps with the PMR-have found compelling evidence for activation patterns in the PCC and precuneus when contrasting faces vs. scenes or unfamiliar vs. familiar faces (Afzalian and Rajimehr, 2021; Kidder et al., 2025; Silson et al., 2019; Woolnough et al., 2020). These patterns closely align with the activation clusters we observed in the PMR for our remote memory condition.

Interestingly, another fMRI study found that the recall of life events (episodic memory), compared to previously learned pictures of various scenes, favored activation in the vmPFC over areas such as the precuneus and PCC, with the opposite pattern observed for the reverse contrast (Chen et al., 2017), suggesting a more nuanced role for the DMN during memory tasks. Although the results from this study do not fully align with our findings, we argue that our data provides further support for specific activation clusters in the PMR that correspond with the retrieval of famous faces and names. In summary, the activation clusters observed in the remote memory condition, particularly involving the PCC, precuneus, and vmPFC, are plausibly explained by their functional engagement in the retrieval of distant episodic memories, whereas the involvement of the ATL regions likely reflect the semantic nature of our task paradigm.

### 4.4 ATL activation vs. HPC absence

In this subsection, we aim to explore the notable absence of the HPC under our memory conditions and elucidate the contributing factors. Our task paradigm apparently led to a systematic prioritized activation of the ATL over the HPC in both conditions. Specifically, in our remote memory condition, the task demands favored activation in regions associated with retrieving distant memories, implying a decreased reliance on the HPC for consolidated memories. However, it is essential to underscore that we cannot dismiss the possibility of a partial contribution from the HPC or its associated regions within the MTL during memory retrieval in our experimental conditions. This becomes especially relevant when considering findings from other studies, where activation clusters in the HPC or MTL were observed during recognition tasks involving famous faces and names (Bernard et al., 2004; Nielson et al., 2010).

At this point, we would like to highlight that, at an uncorrected threshold of *p* < 0.001, we observed small activation clusters in the right HPC during the remote memory condition, as well as in the left parahippocampal region. Additionally, during the recent memory condition, bilateral clusters in the HPC were deactivated at the same threshold. These findings suggest that, although hippocampal involvement tends to decrease over time for remote memories, famous faces may elicit richer memory traces than newly learned ones and therefore engage the HPC and its adjacent regions more strongly (Gilboa and Moscovitch, 2021; Leveroni et al., 2000; Nielson et al., 2010). Some evidence also links hippocampal activation to high-confidence retrieval, which aligns with the higher accuracy observed during the recall of the remote memory condition (Xu et al., 2024). Taken together with the outlined evidence, we argue that, although our analysis did not yield any FWE-corrected results, a partial contribution of the HPC to remote memory retrieval remains likely. An additional explanation for the absence of relevant HPC activity could be linked to our baseline condition, which might have induced associative memory processes, potentially through the repeated presentation of the same faces in our baseline condition. This concern was also raised in a study by Squire et al., highlighting that baseline conditions often lead to notable brain activation, possibly working against the detection of effects in the MTL (Squire and Bayley, 2007). Nevertheless, at this point, we also want to highlight that the absence of MTL activation might contribute to the specificity of our results in the way that for the retrieval of recently and remotely learned face-name pairs the neural correlates found in our study are of primal importance compared to the MTL.

## 5 Conclusion

The bilateral activation in the anterior parts of the STG as subdivision of the ATL observed in the retrieval of recently and remotely associated face-name pairs highlights its role in semantic processing and audiovisual integration. Recent memory retrieval uniquely engaged the anterior insula cortex (aIC). In contrast, remote memory retrieval activated the vmPFC, precuneus and PCC, which are associated with episodic memory and DMN. The present results contribute to our understanding of how semantic and episodic memory networks interplay as a function of memory depending on the retention duration and context in which the association was formed. Our study investigated outcomes in a population of young adults between 19 and 31 years of age. Therefore, it would be interesting to know how age-related neurofunctional changes affect the retrieval of recent and remote memories, particularly within semantic and episodic memory networks. Furthermore, the role of the aIC in AM functioning needs to be elaborated upon further. Thus far, the aIC has been primarily associated with cognitive control as part of the salience network. However, the present results suggest that it also plays a crucial role in AM, especially in the context of recently acquired and retained memories.

## Data Availability

The raw data supporting the conclusions of this article will be made available by the authors, without undue reservation.
